# Role of MicroRNAs in innate neuroprotection mechanisms due to preconditioning of the brain

**DOI:** 10.3389/fnins.2015.00118

**Published:** 2015-04-21

**Authors:** Eva M. Jimenez-Mateos

**Affiliations:** Department of Physiology and Medical Physics, Royal College of Surgeons in IrelandDublin, Ireland

**Keywords:** preconditioning, microRNAs, ischemia, epilepsy, neuroprotection

## Abstract

Insults to the brain that are sub-threshold for damage activate endogenous protective pathways, which can temporarily protect the brain against a subsequent harmful episode. This mechanism has been named as tolerance and its protective effects have been shown in experimental models of ischemia and epilepsy. The preconditioning-stimulus can be a short period of ischemia or mild seizures induced by low doses of convulsant drugs. Gene-array profiling has shown that both ischemic and epileptic tolerance feature large-scale gene down-regulation but the mechanism are unknown. MicroRNAs are a class of small non-coding RNAs of ~20–22 nucleotides length which regulate gene expression at a post-transcriptional level via mRNA degradation or inhibition of protein translation. MicroRNAs have been shown to be regulated after non-harmful and harmful stimuli in the brain and to contribute to neuroprotective mechanisms. This review focuses on the role of microRNAs in the development of tolerance following ischemic or epileptic preconditioning.

## Preconditioning

The concept of preconditioning was first described in a model of heart ischemia. Here, brief ischemic episodes protected against a subsequent ischemic insult (Murry et al., 1986), opening a new way for the treatment of heart diseases. Several preconditioned treatment paradigms have been used in the clinic (including remote preconditioning stimulus) to protect patients against an ischemic insult in heart pathologies (McCafferty et al., 2014).

Preconditioning is an adaptive response where a small dose of a harmful substance protects the brain from a subsequent damaging insult (Murry et al., 1986; Stenzel-Poore et al., 2007; Lin et al., 2008; Dirnagl et al., 2009; Assaf et al., 2011). The fact that any injury to the brain applied below the threshold of cell damage, including seizures, will induce preconditioning and neuroprotection to the brain shows the importance of understanding the mechanism underlying preconditioning and its potential as novel treatment option in brain disorders.

The preconditioning stimulus induces a transient state of protection which is called tolerance. Early work demonstrated that de novo protein synthesis was required for tolerance; this implied regulation of gene expression in a time-specific manner and having an effect only several days after the precondition stimulus (Barone et al., 1998).

Preconditioning can induce neuroprotection over two time-frames: (1) Rapid tolerance, which happens in a short period of time and is independent of protein production and is associated with synapse remodeling (Meller et al., 2008). (2) Delayed (classical) tolerance, which evolves over 1–3 days post-preconditioning and requires de novo protein production which peaks after 3 days and diminishes over the course of the following week (Stenzel-Poore et al., 2007).

### Preconditioning stimuli

Preconditioning can be induced by several stimuli in the brain, including non-injurious ischemia, cortical spreading depression, brief episodes of seizures and low doses of endotoxins (LPS, lipopolysaccharide) (Kitagawa et al., 1990; Simon et al., 1993; Kobayashi et al., 1995; Chen and Simon, 1997; Towfighi et al., 1999). A cross-tolerance phenomenon has been recognized where stimuli and challenge are from different nature. For example, a brief-seizure stimulation or LPS injection can protect against a subsequent ischemic injury (Plamondon et al., 1999; Towfighi et al., 1999).

Data has shown that the reprogramming of genes involved in cellular response to excitotoxic insults plays an important role in the preconditioning process of different tissues (Mirrione et al., 2010). Evidence from genomic studies has demonstrated that diverse stimuli which trigger neuroprotection achieved by preconditioning may share a common process which depends on a fundamental reprogramming of the response to injury, e.g., in ischemia or epileptic tolerance has been shown that 80% of genes are down-regulated. This reprogramming process can induce novel neuroprotective pathways which in turn lead to the synthesis of new proteins changing the molecular and genetic response to subsequent injury (Stenzel-Poore et al., 2004, 2007).

In experimental models of *status epilepticus*, several preconditioning stimuli including bicuculline, electroshock, kindling, and low dose injection of the convulsant kainic acid, has shown to reduce brain damage after *status epilepticus* (Amini et al., 2014). Gene profiling has shown that down-regulation of gene expression is a general mechanism underlying the tolerance process (Borges et al., 2007; Jimenez-Mateos et al., 2008; Meller et al., 2008; Della-Morte et al., 2012), including downregulation of genes involved in ion channels, calcium signaling and excitability (Jimenez-Mateos et al., 2008).

## microRNAs

The term MicroRNA, previously called small temporal RNAs (stRNAs) due to their role in the regulation of developmental timing of *Caernohabditis elegans*, was first introduced in 2001 (Lagos-Quintana et al., 2001; Lau et al., 2001; Lee and Ambros, 2001). Originally microRNAs were defined as small non-coding RNAs (~20–22 nucleotides) that regulate post-transcriptional gene expression in a sequence-specific manner. Since their discovery in *C. elegans*, microRNAs were found to be expressed in invertebrates and vertebrates, including humans, and many show highly-conservation during evolution (Lagos-Quintana et al., 2001).

MiRNAs regulate gene expression via translational inhibition, mRNA degradation or a combination of both mechanisms (O'Carroll and Schaefer, 2013). In the brain, however, miRNA targeting is frequently not associated with reduced mRNA levels of targets (Klein et al., 2007). Almost 50% of all identified miRNAs are expressed in the mammalian brain and there is significant cell- and region-specific distribution reflecting roles in gene expression that direct the functional specialization of neurons and the morphological responses that are required to adapt to their continuously changing activity state (Siegel et al., 2011; O'Carroll and Schaefer, 2013). MiRNAs and their biogenesis components display select localization within neurons, with significant enrichment in dendrites, enabling local, activity-dependent miRNA regulation of protein levels (Lugli et al., 2005, 2008; O'Carroll and Schaefer, 2013). Recent work demonstrated that certain pre-miRs have localization signals which translocate them to the synaptic sites where they are processed into mature miRNA (Bicker et al., 2013).

Most miRNA genes are expressed as a cluster within a single poly-cistronic transcript (Baskerville and Bartel, 2005) and transcribed by RNA polymerase II, resulting in the primary sequence (Cai et al., 2004; Lee et al., 2004). In the nucleus the primary sequence is recognized by the nuclear microprocessor complex containing Drosha and DGCR8 (DiGeorge syndrome critical region 8) which generate the precursor-miRNA (pre-miR) of ~60–70 nt length (Lee et al., 2003). Next, the precursor is exported into the cytoplasm by Exportin-5 in a GTP-dependent manner (Bohnsack et al., 2004). Once in the cytoplasm, microRNAs are further processed by Dicer to a single-strand RNA mature form (Lagos-Quintana et al., 2001; Lau et al., 2001; Lee and Ambros, 2001). Finally, a single strand of the mature miRNA is selected and loaded into the RISC complex and bound to the members of the Argonaute (Ago) protein family. This miRNA-bound-RISC complex is the functional and active form of the miRNA which then targets the mRNA. Binding of a 7–8 nt seed region, usually within the 3′UTR of the mRNA, results in either inhibition of the translation or mRNA degradation. This process requires the activation of de-cap enzymes and removal of the poly-A tail (Kwak and Tomari, 2012).

Several studies have shown the role of miRNAs in the brain by using mainly genetic tools in mice, including conditional or full deletion of biogenesis enzymes in the microRNA pathway. Deletion of DGCR8, which affects the production of the precursor microRNA, results in a reduction in brain size and loss of inhibitory synaptic neurotransmission (Babiarz et al., 2011; Hsu et al., 2012). Conditional deletion of Drosha in neural progenitors did not affect neurogenesis in the developing brain, but did affect differentiation and migration of neurons (Knuckles et al., 2012). Deletion of Dicer from neurons produces severe brain abnormalities, including microencephaly and defects in dendritic arborization in cortex and hippocampus (Davis et al., 2008; De Pietri Tonelli et al., 2008; Babiarz et al., 2011; Dorval et al., 2012). Mice lacking Dicer in astrocytes develop spontaneous seizures and suffer from increased premature mortality (Tao et al., 2011). These data imply that the miRNA biogenesis system is essential for brain development and function. Surprisingly, one study reported that specific deletion of Dicer in the adult mouse forebrain transiently enhanced learning and memory, although these animals later displayed degeneration of neurons in the cortex and hippocampus (Konopka et al., 2010).

Analysis of Argonaute (Ago1–4) proteins, main components of the RISC complex, has given more diverse results (Burroughs et al., 2011). Ago-2 is the most abundant expressed member in various tissues including the brain (Liu et al., 2004) and critical for miRNA-mediated repression of mRNAs (Czech and Hannon, 2011). Deficiency in Ago-2 results in death of mice during early embryogenesis or mid-gestation (Morita et al., 2007). This reflects not only the essential role of Ago2 in embryonic development but perhaps an effect of impaired microRNA generation (Morita et al., 2007). In contrast, studies in conditional mutants showed individual deficiencies in Ago 1, 3, and 4 does not produce obvious effects in mice, suggesting a redundancy among Ago family members (O'Carroll et al., 2007).

## microRNA in ischemia preconditioning

Three different publications show the regulation of microRNAs after an ischemic preconditioning stimulus in parallel (Table 1) (Dharap and Vemuganti, 2010; Lee et al., 2010; Lusardi et al., 2010).

**Table 1:** **MicroRNAs in preconditioning**.

**Preconditioning stimulus**	**microRNAs**	**Target gene**	**References**
Ischemia	Pull ofmiRs	MeCP2	Lusardi et al., 2010
Ischemia	miR-200	PhP2	Lee et al., 2010
Hybernation	miR-200	SUMO	Lee et al., 2012
Epileptic	miR-184	Not determined	McKiernan et al., 2012

Lusardi et al., using a brief ischemic stimulus in mice, showed that preconditioning at the same time up-regulated and down-regulated different microRNAs in the mouse cortex (Lusardi et al., 2010). A common target-gene of the down-regulated microRNAs was the transcriptional regulator MeCP2 (methyl CpG binding protein 2) (Lusardi et al., 2010). Similar results were found when hypertension was used as preconditioning stimulus in rats, where multiple down-regulated microRNAs target MeCP2 (Dharap and Vemuganti, 2010). MeCP2 was first described as a potent transcriptional repressor (Nan, 1998). Mutations in MeCP2 cause Rett syndrome, mental retardation, Angelman syndrome, and autism (Hite et al., 2009; Gonzales and LaSalle, 2010). The up-regulation of MeCP2 via microRNAs corroborated the general gene-repression in tolerance reported before (Lusardi et al., 2010), as increase levels of MeCP2 resulted in a down-regulation of the transcriptome.

In contrast to these studies, Lee et al. showed that neuroprotection achieved by brief ischemic preconditioning stimuli in mice could be due to a family member of miR-200 which targets PHD2 (prolyl hydroxylate 2), which in turn has been involved in the regulation of HIF1α after ischemia (Lee et al., 2010). Up-regulation of miR-200 shortly after the preconditioning stimulus reduced the levels of PHD2 and increased HIF1α. These results were corroborated *in vitro*, where the up-regulation of miR-200 using mimic decreased levels of PHD2 (Lee et al., 2010). Supporting these results, transgenic mice lacking HIF1α exposed to hypoxic preconditioning presented less neuroprotection than control mice after an hypoxia-ischemic insult (Sheldon et al., 2014).

More recently, a model of hibernation, a natural model of tolerance to ischemia, was used to study the role of microRNAs during neuroprotection in the brain (Lee et al., 2012). During torpor stages, hibernating animals lower their energy consumption, blood flow, and body temperature to an otherwise lethal level which is similar to levels during an ischemic insult (Lee et al., 2012). Microarray analysis from ground squirrel brains were performed during the active and the torpor phase and 405 microRNAs were different between both stages, with the family of miR-200 being the most representative, consistent with Lee et al. (2010). In this study the authors suggest that miR-200 family could regulate members of the SUMO family which is involved in the ubiquitin regulation and protein degradation, thereby explaining the reduction in protein levels during tolerance (Lee et al., 2012).

## microRNA in epileptic preconditioning

Similar to ischemia, several studies analyzed the role of microRNAs after a preconditioning-seizure stimuli (Table 1 and Hatazaki et al., 2007; Kretschmann et al., 2015).

McKiernan and colleagues by using low dose of systemic KA in mice showed a de-regulation of microRNA after epileptic preconditioning. Here, a general up-regulation of microRNAs was the main response in preconditioned mice compared to control mice (McKiernan et al., 2012). Whereas 9 microRNAs were uniquely present in the preconditioning group, 39 microRNAs showed increase and 6 reduced levels in the pre-conditioning group. From the up-regulated microRNAs, 25 of the 39 were significantly regulated (McKiernan et al., 2012). Between the up-regulated microRNA group, the highest levels were found for miR-184. Inhibition of miR-184 *in vivo* resulted in neuronal death after a normally non-damaging preconditional stimulus (McKiernan et al., 2012) and an increase in neurodegeneration during the tolerant state after status epilepticus (damaging injury stimulation). Together, these results show a neuroprotective role of miR-184 during preconditioning (McKiernan et al., 2012).

Similar results have been found in acute seizure models not associated with neuronal death (Kretschmann et al., 2015). Kretschman and colleagues showed that 6 h after the induction of a single seizure via electrical stimulation up-regulation of microRNAs was the main response. 146 microRNAs were de-regulated, with almost 140 microRNAs being up-regulated and only few of them down-regulated (Kretschmann et al., 2015).

In a more detailed study, the expression levels of microRNAs were analyzed after electroshock stimulation, showing that microRNA levels follow three distinct patterns after non-damage neuronal activity (Eacker et al., 2011). MicroRNA regulation can be clustered in three distinct groups: Class 1, microRNAs which progressively increase their expression levels after electrical stimulation, including miR-134; Class 2, microRNAs which are strongly up-regulated in the first hours after stimulation with levels decreasing back to normal rapidly; Class 3, microRNAs that increase initially but decrease at a later time-point (12 or 24 h after the stimulation) (Eacker et al., 2011). This study shows the specific response of microRNAs after brain stimulation, and its possible role in the different tolerant-stages.

## Targeting microRNAs on brain

Several approaches for manipulating miRNA levels in neuronal cells *in vitro* and *in vivo* have been described, including recent applications of miRNA antisense oligonucleotides, miRNA gene knockout and miRNA sponges in neuronal cells. The efficiency of microRNAs inhibitors has been reported several times, inhibition of miR184 show a loss in the preconditioning neuroprotection without any toxicity (McKiernan et al., 2012). In contrast, no many approaches have shown over-expression of microRNAs in the brain, possible related with the toxic effect increase levels of RNA in the brain. The study of new drugs which target microRNAs in the central nervous system will be one of the main fields of future investigations.

## Conclusions

Studying the molecular mechanism underlying the innate neuroprotection can help to understand how the brain protects itself against a damaging insult. Analysis of tolerant states has shown that a general down-regulation of genes is the main response in the brain, independently of the original insult, ischemic, or seizures. MicroRNAs are master regulators of gene-expression, its regulation after the preconditioning stimulus could be causative of the general suppression of gene-expression and being a good candidate for future target-therapy. Still several questions are remaining: Can we regulate microRNAs in the brain? Systemic administration of microRNA mimics or microRNA inhibitors will be necessary. Can we regulate microRNA expression in a time dependent manner? Tolerant is a time-dependent effect which only last for several days, a longer inhibition or over-expression of microRNAs could be toxic for the brain.

### Conflict of interest statement

The author declares that the research was conducted in the absence of any commercial or financial relationships that could be construed as a potential conflict of interest.
